# Supplementary material for: Risk factors for treatment failure in women with uncomplicated lower urinary tract infection
Source: PLoS One. 2021 Aug 31;16(8):e0256464. doi: 10.1371/journal.pone.0256464 (PMC8407559; doi:10.1371/journal.pone.0256464)
Supplement: S1 Appendix — (DOCX) [file pone.0256464.s001.docx]

Martischang R, Godycki- Ćwirko M, Anna Kowalczyk, MS, et al. Risk factors for treatment failure in women with uncomplicated lower urinary tract infection.

**Supplementary Appendix 1.** Definition of variables originally collected

**Figure S1.** Variation in clinical and microbiologic failure across age and treatment groups in the E. coli-related UTI subcohort

**Figure S2.** Results of various models based on Bayesian model averaging for predicting treatment failure in the microbiologically confirmed cohort

**Figure S3.** Results of various models based on Bayesian model averaging for predicting treatment failure in the PPP population

**Figure S4.** Results of various models based on Bayesian model averaging for predicting treatment failure in the E. coli cohort

**Figure S5.** Log-linearity graph for clinical failure

**Table S1.** Population characteristics in the per-protocol population (PPP) regarding clinical failure

**Table S2.** Population characteristics in the microbiologically-confirmed cohort regarding clinical failure

**Table S3.** Population characteristics in the *E. coli* cohort regarding clinical failure

**Table S4.** Population characteristics in the microbiologically-confirmed cohort regarding microbiological failure

**Table S5.** Population characteristics in the *E. coli* cohort regarding microbiological failure

**Table S6**. Population characteristics among included and excluded participants (with undetermined results or not adhering to the initial trial’s protocol)

**Table S7.** Multivariate models for clinical failure in the *E. coli* subcohort

**Table S8.** Multivariate models for microbiologic failure in the *E. coli* subcohort

**Table S9.** Multivariate models for clinical failure in the PPP cohort

**Table S10.** Multivariate models for clinical failure in the microbiologically confirmed cohort with addition of microbiological exposure

**Table S11.** Multivariate models for microbiological failure in the microbiologically confirmed cohort with addition of microbiological exposure

**Table S12.** Multivariate logistic regression model assessing negative culture at baseline

**STROBE Statement**—Checklist of items that should be included in reports of case-control studies

**Supplementary References**

This appendix has been provided by the authors to provide readers additional information about this study.

**Supplementary Appendix 1. Definition of variables originally collected**

Variables originally considered in the clinical trial and included in this secondary analysis are recruitment site, age, risk factors for multidrug-resistant-organism (MDRO) carriage, history of recurrent UTI, and antibiotic treatment received. Patient history was originally collected, and pertinent comorbidities were extracted for our study (diabetes mellitus, history of UTI with or without pyelonephritis). Symptoms and/or signs include dysuria, frequency, urgency, suprapubic tenderness, flank pain, nausea, vomiting, gross hematuria, subjective fever, chills, and presence of leucocytes on dipstick analysis.

As defined previously, risk factors for MDRO carriage included systemic antibiotic exposure in the previous 12 months, hospitalization in an acute- or long-term-care-centre in the previous 12 months, healthcare-associated infection, carriage of resistant organisms in the previous 12 months, and travel/residency in the past year in endemic countries. Positive cultures were originally defined as 10^3^ cfu/mL or more of at least 1 bacterium detected. Resistance was tested in Poland and Israel according to Clinical and Laboratory Standards Institute, and in Switzerland, according to the European Committee on Antimicrobial Susceptibility Testing breakpoints.^1^

**Supplementary Figures**


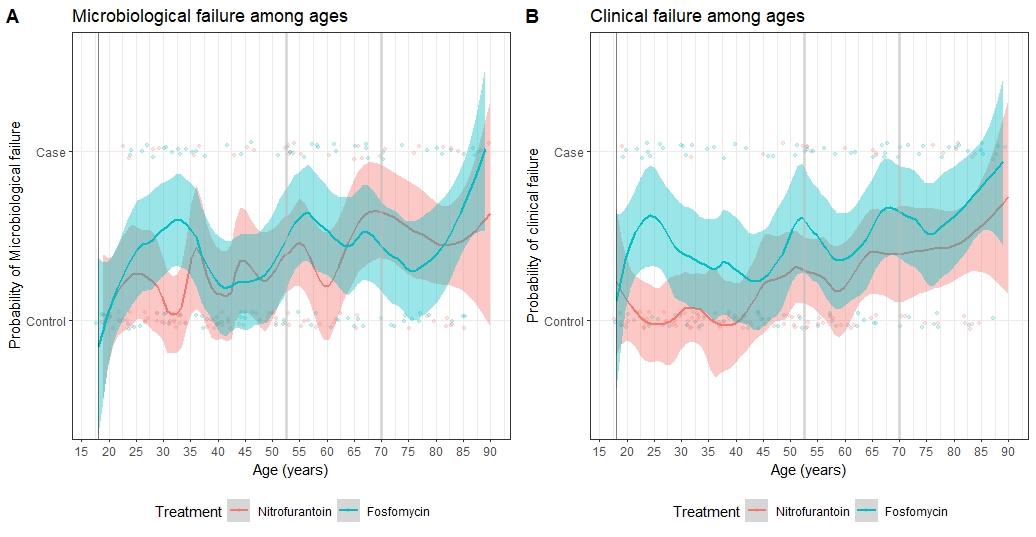


Figure S1. Variation in clinical and microbiologic failure across age and treatment groups in the E. coli-related UTI subcohort.

| **Bayesian model averaging of co-variate for clinical failure**  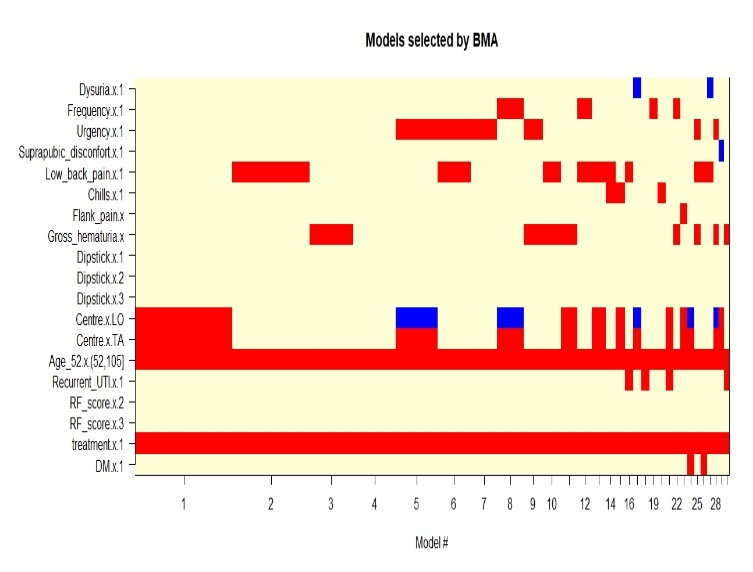 | **Bayesian model averaging of co-variate for microbiologic failure**  **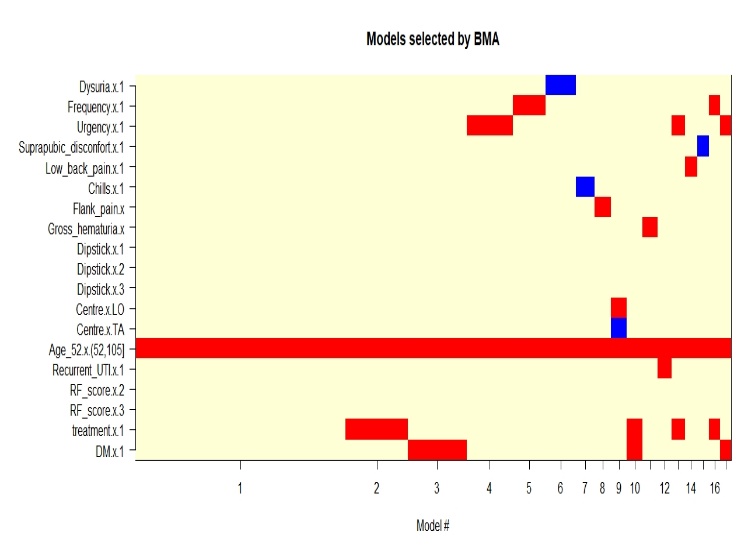** |
| --- | --- |

Supplementary Figure 2. Results of various models based on Bayesian model averaging for predicting treatment failure in the microbiologically confirmed cohort.

Footnote to figure S2:

In red: positive coefficient, in blue: negative coefficient obtained from various logistic regression models


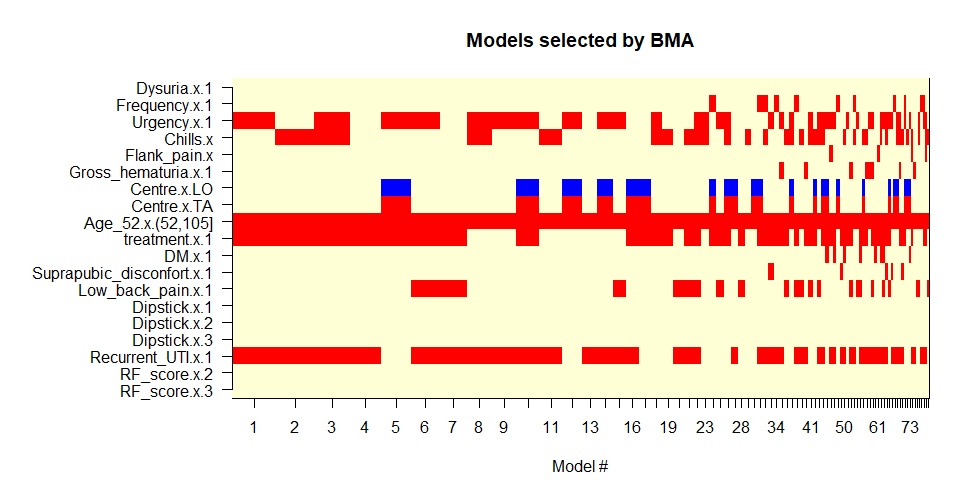


Figure S3. Results of various models based on Bayesian model averaging for predicting treatment failure in the PPP population

Footnote to figure S3:

In red: positive coefficient, in blue: negative coefficient obtained from various logistic regression models

| **Bayesian model averaging of co-variate for clinical failure**  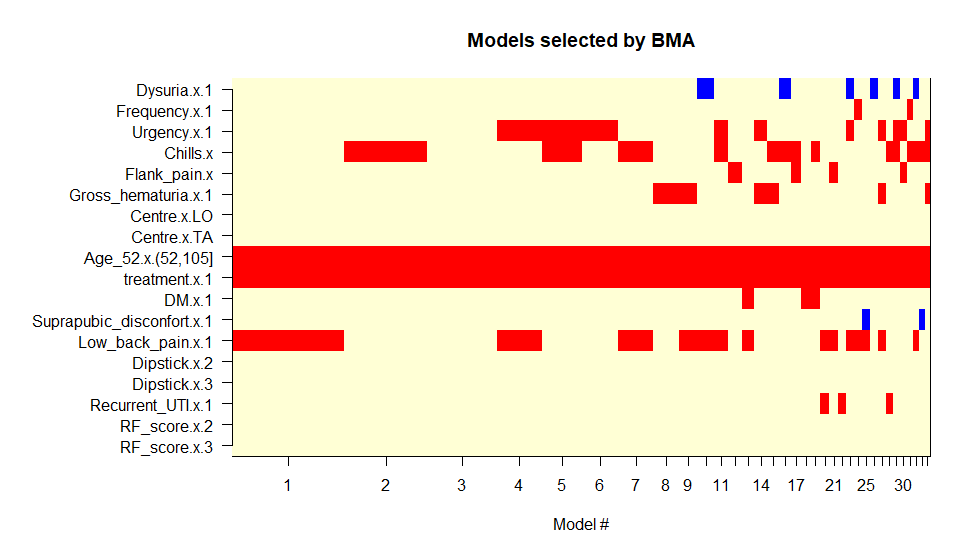 | **Bayesian model averaging of co-variate for microbiologic failure**  **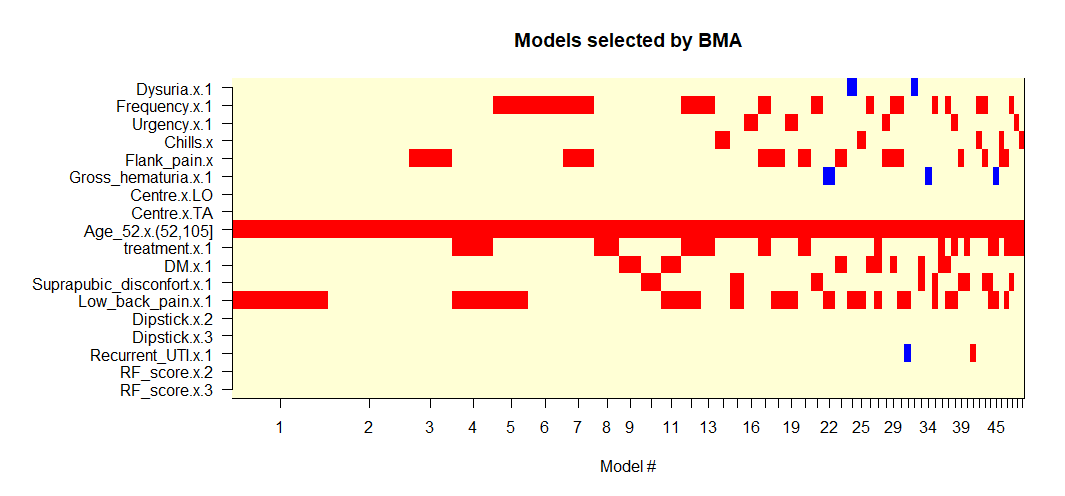** |
| --- | --- |

Figure S4. Results of various models based on Bayesian model averaging for predicting treatment failure in the E. coli cohort

Footnote to figure S4:

In red: positive coefficient, in blue: negative coefficient obtained from various logistic regression models

| 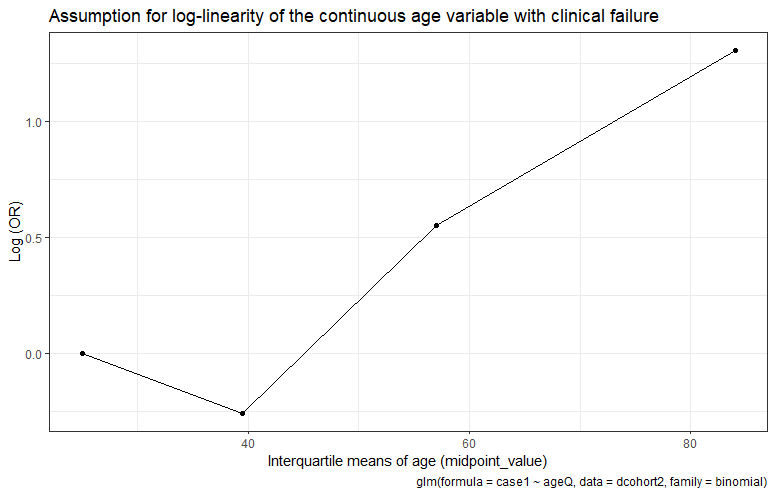 |  |
| --- | --- |

Figure S5. Log-linearity graph for clinical failure

**Supplementary Tables**

Table S1. Population characteristics in the per-protocol population (PPP) regarding clinical failure

|  | **Clinical Failure** | |  |
| --- | --- | --- | --- |
|  | **Cases (n= 152)** | **Controls (n= 307)** | **P value*** |
| **Age ≥52 years (%)** | **87 (57.2)** | **102 (33.2** | **<0.001** |
| Dysuria (%) | 113 (74.3) | 237 (77.2) | 0.58 |
| Frequency (%) | 140 (92.1) | 267 (87) | 0.14 |
| **Urgency (%)** | **130 (85.5)** | **231 (75.2)** | **0.02** |
| Suprapubic disconfort (%) | 79 (52) | 148 (48.2) | 0.51 |
| Gross hematuria (%) | 26 (17.1) | 44 (14.3) | 0.52 |
| Flank pain (%) | 21 (13.8) | 30 (9.8) | 0.25 |
| **Chills (%)** | **26 (17.1)** | **25 (8.1)** | **0.01** |
| **Reccurent UTI (%)** | **35 (23)** | **35 (11.4)** | **0.002** |
| **Diabetes mellitus (%)** | **22 (14.5)** | **20 (6.5)** | **0.01** |
| **Fosfomycin (%)** | **91 (59.9)** | **138 (45)** | **0.004** |
| **Inclusion in Geneva (%)** | 44 (28.9) | 115 (37.5) | **0.002** |
| **Inclusion in Lodz (%)** | 57 (37.5) | 134 (43.6) |  |
| **Inclusion in Tel-Aviv (%)** | 51 (33.6) | 58 (18.9) |  |
| Risk score for resistance = 0 (%) | 15 (9.9) | 40 (13) | 0.3 |
| Risk score for resistance = 1 (%) | 115 (75.7) | 211 (68.7) |  |
| Risk score for resistance = 2 (%) | 22 (14.5) | 56 (18.2) |  |
| Nitrites on dipstick (%) | 5 (3.3) | 10 (3.3) |  |
| Leukocytes on dipstick (%) | 84 (55.3) | 165 (53.7) |  |
| Both on dipstick (%) | 63 (41.4) | 130 (42.3) |  |

*P value by X^2^ test.

Table S2. Population characteristics in the microbiologically-confirmed cohort regarding clinical failure

|  | **Clinical Failure** | |  |
| --- | --- | --- | --- |
|  | **Case (n= 126)** | **Control (n= 214)** | **P value*** |
| **Age ≥52 years (%)** | **75 (59.5)** | **74 (34.6)** | **<0.001** |
| Dysuria (%) | 94 (74.6) | 174 (81.3) | 0.19 |
| Frequency (%) | 116 (92.1) | 185 (86.4) | 0.16 |
| **Urgency (%)** | **106 (84.1)** | **158 (73.8)** | **0.04** |
| Suprapubic disconfort (%) | 60 (47.6) | 102 (47.7) | 1 |
| Gross hematuria (%) | 23 (18.3) | 32 (15) | 0.52 |
| Flank pain (%) | 16 (12.7) | 17 (7.9) | 0.21 |
| **Chills (%)** | **21 (16.7)** | **19 (8.9)** | **0.05** |
| Reccurent UTI (%) | 24 (19) | 27 (12.6 ) | 0.15 |
| Diabetes mellitus (%) | 18 (14.3) | 16 (7.5) | 0.07 |
| **Fosfomycin (%)** | **78 (61.9)** | **87 (40.7)** | **<0.001** |
| **Inclusion in Geneva (%)** | **43 (34.1)** | **104 (48.6)** | **0.0015** |
| **Inclusion in Lodz (%)** | **49 (38.9)** | **83 (38.8)** | **0.0015** |
| **Inclusion in Tel-Aviv (%)** | **34 (27)** | **27 (12.6)** | **0.0015** |
| **Risk score for resistance = 0 (%)** | **15 (11.9)** | **36 (16.8)** | **0.05** |
| **Risk score for resistance = 1 (%)** | **94 (74.6)** | **132 (61.7)** | **0.05** |
| **Risk score for resistance = 2 (%)** | **17 (13.5)** | **46 (21.5** | **0.05** |
| Nitrites on dipstick (%) | 3 (2.4) | 8 (3.7) | 0.51 |
| Leukocytes on dipstick (%) | 64 (50.8) | 97 (45.3) | 0.51 |
| Both on dipstick (%) | 59 (46.8) | 107 (50) | 0.51 |

*P value by X^2^test.

Table S3. Population characteristics in the E. coli cohort regarding clinical failure

|  | **Clinical Failure** | |  |
| --- | --- | --- | --- |
|  | **Cases (n= 70)** | **Controls (n= 134)** | **P value*** |
| **Age ≥52 years (%)** | **43 (61.4)** | **41 (30.6)** | **<0.001** |
| Dysuria (%) | 52 (74.3) | 112 (83.6) | 0.16 |
| Frequency (%) | 65 (92.9) | 123 (91.8) | 1 |
| Urgency (%) | 62 (88.6) | 104 (77.6) | 0.09 |
| Suprapubic disconfort (%) | 30 (42.9) | 61 (45.5) | 0.83 |
| Gross hematuria (%) | 16 (22.9) | 23 (17.2) | 0.43 |
| Flank pain (%) | 8 (11.4) | 7 (5.2) | 0.18 |
| Chills (%) | 12 (17.1) | 10 (7.5) | 0.06 |
| Reccurent UTI (%) | 14 (20) | 18 (13.4) | 0.31 |
| **Diabetes mellitus (%)** | **12 (17.1)** | **9 (6.7)** | **0.04** |
| **Fosfomycin (%)** | **51 (72.9)** | **55 (41)** | **<0.001** |
| Inclusion in Geneva (%) | 29 (41.4) | 67 (50) | 0.32 |
| Inclusion in Lodz (%) | 32 (45.7) | 57 (42.5) | 0.32 |
| Inclusion in Tel-Aviv (%) | 9 (12.9) | 10 (7.5) | 0.32 |
| Risk score for resistance = 0 (%) | 10 (14.3) | 20 (14.9) | 0.36 |
| Risk score for resistance = 1 (%) | 49 (70) | 82 (61.2) | 0.36 |
| Risk score for resistance = 2 (%) | 11 (15.7) | 32 (23.9) | 0.36 |
| Nitrites on dipstick (%) | 1 (1.4) | 4 (3) | 0.74 |
| Leukocytes on dipstick (%) | 29 (41.4) | 51 (38.1) | 0.74 |
| Both on dipstick (%) | 40 (57.1) | 79 (59) | 0.74 |

*P value by X^2^test.

Table S4. Population characteristics in the microbiologically-confirmed cohort regarding microbiological failure

|  | **Microbiological Failure** | |  |
| --- | --- | --- | --- |
|  | **Case (n= 102)** | **Control (n= 220)** | **P value*** |
| **Age (+/-52 years)** | **64 (62.7)** | **82 (37.3)** | **<0.001** |
| Dysuria (%) | 74 (72.5) | 181 (82.3) | 0.06 |
| Frequency (%) | 95 (93.1) | 192 (87.3) | 0.17 |
| Urgency (%) | 85 (83.3) | 167 (75.9) | 0.17 |
| Suprapubic disconfort (%) | 43 (42.2) | 104 (47.3) | 0.46 |
| Gross hematuria (%) | 11 (10.8) | 38 (17.3) | 0.18 |
| Flank pain (%) | 12 (11.8) | 18 (8.2) | 0.41 |
| Chills (%) | 12 (11.8) | 22 (10) | 0.78 |
| Reccurent UTI (%) | 17 (16.7) | 32 (14.5) | 0.74 |
| **Diabetes Mellitus (%)** | **18 (17.6)** | **16 (7.3)** | **0.01** |
| Fosfomycin (%) | 58 (56.9) | 99 (45) | 0.06 |
| **Inclusion in Geneva (%)** | **38 (37.3)** | **108 (49.1)** | **0.004** |
| **Inclusion in Lodz (%)** | **55 (53.9)** | **77 (35)** | **0.004** |
| **Inclusion in Tel-Aviv (%)** | **9 (8.8)** | **35 (15.9)** | **0.004** |
| Risk score for resistance = 0 (%) | 14 (13.7) | 36 (16.4) | 0.79 |
| Risk score for resistance = 1 (%) | 69 (67.6) | 141 (64.1) | 0.79 |
| Risk score for resistance = 2 (%) | 19 (18.6) | 43 (19.5) | 0.79 |
| **Nitrites on dipstick (%)** | **4 (3.9)** | **7 (3.2)** | **0.03** |
| **Leukocytes on dipstick (%)** | **35 (34.3)** | **111 (50.5)** | **0.03** |
| **Both on dipstick (%)** | **63 (61.8)** | **100 (45.5)** | **0.03** |

*P value by X^2^test.

Table S5. Population characteristics in the E. coli cohort regarding microbiological failure

|  | **Microbiological Failure** | |  |
| --- | --- | --- | --- |
|  | **Case (n= 102)** | **Control (n= 220)** | **P value*** |
| **Age (+/-52 years)** | **43 (60.6)** | **41 (32)** | **0.00017** |
| Dysuria (%) | 54 (76.1) | 106 (82.8) | 0.34 |
| Frequency (%) | 69 (97.2) | 115 (89.8) | 0.11 |
| Urgency (%) | 62 (87.3) | 101 (78.9) | 0.2 |
| Suprapubic disconfort (%) | 34 (47.9) | 53 (41.4) | 0.46 |
| Gross hematuria (%) | 10 (14.1) | 26 (20.3) | 0.37 |
| **Flank pain (%)** | **9 (12.7)** | **5 (3.9)** | **0.04** |
| Chills (%) | 10 (14.1) | 10 (7.8) | 0.24 |
| Reccurent UTI (%) | 12 (16.9) | 20 (15.6) | 0.97 |
| **Diabetes Mellitus (%)** | **13 (18.3)** | **8 (6.2)** | **0.02** |
| Fosfomycin (%) | 44 (62) | 61 (47.7) | 0.07 |
| Inclusion in Geneva (%) | 29 (40.8) | 66 (51.6) | 0.35 |
| Inclusion in Lodz (%) | 36 (50.7) | 53 (41.4) | 0.35 |
| Inclusion in Tel-Aviv (%) | 6 (8.5) | 9 (7) | 0.35 |
| Risk score for resistance = 0 (%) | 11 (15.5) | 18 (14.1) | 0.92 |
| Risk score for resistance = 1 (%) | 46 (64.8) | 82 (64.1) | 0.92 |
| Risk score for resistance = 2 (%) | 14 (19.7) | 28 (21.9) | 0.92 |
| Nitrites on dipstick (%) | 1 (1.4) | 4 (3.1) | 0.16 |
| Leukocytes on dipstick (%) | 22 (31) | 55 (43) | 0.16 |
| Both on dipstick (%) | 48 (67.6) | 69 (53.9) | 0.16 |

*P value by X^2^test.

Table S6. Population characteristics among included and excluded participants (with undetermined results or not adhering to the initial trial’s protocol)

|  | **Included (n= 459)** | **Excluded (n= 54)** | **P value*** |
| --- | --- | --- | --- |
| Clinical failure (%) | 152 ( 33.1 % ) | 8 ( 72.7 % )** | 0.01 |
| Microbiologic failure (%) | 102 ( 31.7 % ) | 4 ( 25% )** | 0.77 |
| Age (+/-52 years) | 189 ( 41.2 % ) | 18 ( 34 % ) | 0.39 |
| Dysuria (%) | 350 ( 76.3 % ) | 44 ( 83 % ) | 0.35 |
| Frequency (%) | 407 ( 88.7 % ) | 39 ( 73.6 % ) | **<0.01** |
| Urgency (%) | 361 ( 78.6 % ) | 38 ( 71.7 % ) | 0.33 |
| Suprapubic disconfort (%) | 227 ( 49.5 % ) | 38 ( 71.7 % ) | **<0.01** |
| Gross hematuria (%) | 70 ( 15.3 % ) | 12 ( 22.6 % ) | 0.23 |
| Flank pain (%) | 51 ( 11.1 % ) | 10 ( 18.9 % ) | 0.15 |
| Chills (%) | 51 ( 11.1 % ) | 6 ( 11.3 % ) | 1.00 |
| Recurrent UTI (%) | 70 ( 15.3 % ) | 14 ( 25.9 % ) | 0.07 |
| Diabetes Mellitus (%) | 42 ( 9.2 % ) | 3 ( 5.6 % ) | 0.53 |
| Treatment received (%) | 229 ( 49.9 % ) | 29 ( 53.7 % ) | 0.70 |
| Inclusion in Geneva (%) | 159 ( 34.6 % ) | 27 ( 50 % ) | **<0.01** |
| Inclusion in Lodz (%) | 191 ( 41.6 % ) | 9 ( 16.7 % ) | **<0.01** |
| Inclusion in Tel-Aviv (%) | 109 ( 23.7 % ) | 18 ( 33.3 % ) | **<0.01** |
| Risk score for resistance = 0 (%) | 55 ( 12 % ) | 7 ( 13.2 % ) | 0.93 |
| Risk score for resistance = 1 (%) | 326 ( 71 % ) | 36 ( 67.9 % ) |  |
| Risk score for resistance = 2 (%) | 66 ( 14.4 % ) | 9 ( 17 % ) |  |
| Risk score for resistance = 3 (%) | 12 ( 2.6% ) | 1 ( 1.9% ) |  |
| Nitrites on dipstick (%) | 15 ( 3.3 % ) | 2 ( 3.8 % ) | **0.02** |
| Leukocytes on dipstick (%) | 249 ( 54.2 % ) | 34 ( 64.2 % ) |  |
| Both on dipstick (%) | 193 ( 42 % ) | 16 ( 30.2 % ) |  |

*P value by X^2^ test.

** Patients with missing events or undetermined results were excluded

Table S7. Multivariate models for clinical failure in the E. coli subcohort.

|  | Clinical failure | | | |
| --- | --- | --- | --- | --- |
| Terms | **Odds-Ratio** | **2.5% CI** | **97.5% CI** | **P value** |
| Age [0;52.5] | *Reference* | | | |
| Age [52.5;105] | 4.03 | 2.05 | 8.18 | **<0.001***** |
| Nitrofurantoin | *Reference* | | | |
| Fosfomycin | 4.12 | 2.14 | 8.24 | **<0.001***** |
| Centre (GE) | *Reference* | | | |
| Centre (LO) | 0.87 | 0.42 | 1.78 | 0.71 |
| Centre (TA) | 2.76 | 0.87 | 8.74 | 0.08 |

Table S8. Multivariate models for microbiologic failure in the E. coli subcohort.

| Terms | Odds ratio | 2.5% CI | 97.5% CI | P value |
| --- | --- | --- | --- | --- |
| Nitrofurantoin | *Reference* |  |  |  |
| Fosfomycin | 1.72 | 0.94 | 3.21 | 0.083 |
| Age [0;52.5] | *Reference* |  |  |  |
| Age [52.5;105] | 3.08 | 1.64 | 5.87 | **<0.001***** |
| Centre (GE) | *Reference* |  |  |  |
| Centre (LO) | 1.14 | 0.59 | 2.21 | 0.694 |
| Centre (TA) | 1.41 | 0.41 | 4.56 | 0.569 |

Table S9. Multivariate models for clinical failure in the PPP cohort.

| Terms | Odds ratio | 2.5% CI | 97.5% CI | P value |
| --- | --- | --- | --- | --- |
| Nitrofurantoin | *Reference* |  |  |  |
| Fosfomycin | 1.77 | 1.18 | 2.68 | **0.006**** |
| Age [0;52.5] | *Reference* |  |  |  |
| Age [52.5;105] | 3.07 | 2.01 | 4.75 | **<0.001***** |
| Centre (GE) | *Reference* |  |  |  |
| Centre (LO) | 0.82 | 0.49 | 1.34 | 0.425 |
| Centre (TA) | 2.29 | 1.34 | 3.92 | **0.002**** |

Table S10. Multivariate models for clinical failure in the microbiologically-confirmed cohort with addition of microbiological exposure

| Terms | Odds ratio | 2.5% CI | 97.5% CI | P value |
| --- | --- | --- | --- | --- |
| Nitrofurantoin | *Reference* |  |  |  |
| Fosfomycin | 2.43 | 1.50 | 3.97 | **<0.001***** |
| Age [0;52.5] | *Reference* |  |  |  |
| Age [52.5;105] | 2.92 | 1.78 | 4.87 | **<0.001***** |
| Centre (GE) | *Reference* |  |  |  |
| Centre (LO) | 1.05 | 0.60 | 1.83 | 0.8 |
| Centre (TA) | 3.07 | 1.56 | 6.13 | **0.001**** |
| Resistance to the study drug | 1.42 | 0.50 | 3.94 | 0.5 |
| Presence of *E.coli* at baseline | 0.96 | 0.57 | 1.62 | 0.9 |

Table S11. Multivariate models for microbiological failure in the microbiologically-confirmed cohort with addition of microbiological exposure

| Terms | Odds ratio | 2.5% CI | 97.5% CI | P value |
| --- | --- | --- | --- | --- |
| Nitrofurantoin | *Reference* |  |  |  |
| Fosfomycin | 1.57 | 0.95 | 2.62 | 0.08 |
| Age [0;52.5] | *Reference* |  |  |  |
| Age [52.5;105] | 2.61 | 1.56 | 4.40 | **<0.001***** |
| Centre (GE) | *Reference* |  |  |  |
| Centre (LO) | 1.64 | 0.95 | 2.84 | 0.08 |
| Centre (TA) | 0.75 | 0.30 | 1.74 | 0.5 |
| Resistance to the study drug | 1.88 | 0.61 | 5.52 | 0.2 |
| Presence of *E.coli* at baseline | 1.75 | 1.01 | 3.10 | 0.05 |

Table S12. Multivariate logistic regression model assessing negative culture at baseline.

|  | **Clinical failure** | | | |
| --- | --- | --- | --- | --- |
| **Terms** | **Odds-Ratio** | **2.5% CI** | **97.5% CI** | **P value** |
| Age [0;52.5] | *Reference* | | | |
| Age [52.5;105] | 2.88 | 1.84 | 4.56 | **<0.001***** |
| Nitrofurantoin | *Reference* | | | |
| Fosfomycin | 2.03 | 1.32 | 3.17 | **<0.001***** |
| Centre (GE) | *Reference* | | | |
| Centre (LO) | 1.05 | 0.62 | 1.76 | 0.86 |
| Centre (TA) | 3.04 | 1.67 | 5.59 | **<0.001***** |
| Culture negative at baseline | 0.23 | 0.11 | 0.44 | **<0.001***** |

**STROBE Statement—Checklist of items that should be included in reports of case-control studies**

|  | Item No | Recommendation |  |
| --- | --- | --- | --- |
| **Title and abstract** | 1 | (*a*) Indicate the study’s design with a commonly used term in the title or the abstract | Title page |
|  |  | (*b*) Provide in the abstract an informative and balanced summary of what was done and what was found | P.2 |
| Introduction | | |  |
| Background/rationale | 2 | Explain the scientific background and rationale for the investigation being reported | P.3-4 |
| Objectives | 3 | State specific objectives, including any prespecified hypotheses | P.3 |
| Methods | | |  |
| Study design | 4 | Present key elements of study design early in the paper | P.4 |
| Setting | 5 | Describe the setting, locations, and relevant dates, including periods of recruitment, exposure, follow-up, and data collection | P.4 |
| Participants | 6 | (*a*) Give the eligibility criteria, and the sources and methods of case ascertainment and control selection. Give the rationale for the choice of cases and controls | P.4 |
|  |  | (*b*) For matched studies, give matching criteria and the number of controls per case | N/A |
| Variables | 7 | Clearly define all outcomes, exposures, predictors, potential confounders, and effect modifiers. Give diagnostic criteria, if applicable | P.4 |
| Data sources/ measurement | 8* | For each variable of interest, give sources of data and details of methods of assessment (measurement). Describe comparability of assessment methods if there is more than one group | P.4 |
| Bias | 9 | Describe any efforts to address potential sources of bias | P.4-5 |
| Study size | 10 | Explain how the study size was arrived at | N/A |
| Quantitative variables | 11 | Explain how quantitative variables were handled in the analyses. If applicable, describe which groupings were chosen and why | P.5 |
| Statistical methods | 12 | (*a*) Describe all statistical methods, including those used to control for confounding | P.5 |
|  |  | (*b*) Describe any methods used to examine subgroups and interactions | P.5 |
|  |  | (*c*) Explain how missing data were addressed | P.4 |
|  |  | (*d*) If applicable, explain how matching of cases and controls was addressed | N/A |
|  |  | (*e*) Describe any sensitivity analyses | P.5 |
| Results | | |  |
| Participants | 13* | (a) Report numbers of individuals at each stage of study—eg numbers potentially eligible, examined for eligibility, confirmed eligible, included in the study, completing follow-up, and analysed | P.6 |
|  |  | (b) Give reasons for non-participation at each stage | P.6 |
|  |  | (c) Consider use of a flow diagram | Fig.1 |
| Descriptive data | 14* | (a) Give characteristics of study participants (eg demographic, clinical, social) and information on exposures and potential confounders | Table 1 |
|  |  | (b) Indicate number of participants with missing data for each variable of interest | N/A |
| Outcome data | 15* | Report numbers in each exposure category, or summary measures of exposure | Table 1 |
| Main results | 16 | (*a*) Give unadjusted estimates and, if applicable, confounder-adjusted estimates and their precision (eg, 95% confidence interval). Make clear which confounders were adjusted for and why they were included | Table 2, P.6-7 |
|  |  | (*b*) Report category boundaries when continuous variables were categorized | Table 2, P.6-7 |
|  |  | (*c*) If relevant, consider translating estimates of relative risk into absolute risk for a meaningful time period | N/A |

| Other analyses | 17 | Report other analyses done—eg analyses of subgroups and interactions, and sensitivity analyses | P.2 |
| --- | --- | --- | --- |
| Discussion | | |  |
| Key results | 18 | Summarise key results with reference to study objectives | P.6 |
| Limitations | 19 | Discuss limitations of the study, taking into account sources of potential bias or imprecision. Discuss both direction and magnitude of any potential bias | P.6 |
| Interpretation | 20 | Give a cautious overall interpretation of results considering objectives, limitations, multiplicity of analyses, results from similar studies, and other relevant evidence | P.7 |
| Generalisability | 21 | Discuss the generalisability (external validity) of the study results | P.7 |
| Other information | | |  |
| Funding | 22 | Give the source of funding and the role of the funders for the present study and, if applicable, for the original study on which the present article is based | P.8 |

*Give information separately for cases and controls.

**Note:** An Explanation and Elaboration article discusses each checklist item and gives methodological background and published examples of transparent reporting. The STROBE checklist is best used in conjunction with this article (freely available on the Web sites of PLoS Medicine at http://www.plosmedicine.org/, Annals of Internal Medicine at http://www.annals.org/, and Epidemiology at http://www.epidem.com/). Information on the STROBE Initiative is available at http://www.strobe-statement.org.

**Supplementary References**

1. Huttner, A. *et al.* Effect of 5-Day Nitrofurantoin vs Single-Dose Fosfomycin on Clinical Resolution of Uncomplicated Lower Urinary Tract Infection in Women: A Randomized Clinical Trial. *JAMA* **319**, 1781 (2018).
