# Supplementary material for: Risk factors for treatment failure in women with uncomplicated lower urinary tract infection
Source: PLoS One. 2021 Aug 31;16(8):e0256464. doi: 10.1371/journal.pone.0256464 (PMC8407559; doi:10.1371/journal.pone.0256464)
Supplement: Attachment — Submitted filename: Response to Reviewers.docx [file pone.0256464.s002.docx]

**PONE-D-21-14320 (“Risk factors for treatment failure in women with uncomplicated lower urinary tract infection”)**

**Reply to reviewers:**

On behalf of the authors, we would like to thank the reviewers for their careful reading and valuable, in-depth comments. We provide point-by-point responses below. Furthermore, we have ensured that our manuscript meets PLOS ONE’s style requirements, and have made the minimal anonymized data set available to the best of our ability on Zenodo (<https://zenodo.org/>, DOI: 10.5281/zenodo.5024262). We used data collected from Geneva (Switzerland), Lodz (Poland), and Tel Aviv (Israel). By Swiss and Polish laws, there is no restriction to share anonymized information. However, the situation is more complicated for Israeli data. Additional approvals from both the Ethics Committee and the Data Privacy Committee would be required, and in the experience of our co-investigator, Prof. Leonard Leibovici, are unlikely to be granted, as his organization does not typically allow ‘placing its patients’ data into public view’, even when anonymized. We will thus be able to provide only the Swiss and Polish dataset; we hope that you will deem this sufficient. If individual researchers want to recycle the complete dataset, we will offer them to contact us any time to find an appropriate solution. After inclusion of multiple details in the revised manuscript as requested by the reviewers, the MS length has increased to 2’172 words (text only), despite all attempts to shorten the main text.

# Reviewer #1

**General comment:**

The article has been well written and provides evidence for risk factors for clinical and microbiological failure of treatment in a relatively large population of women with lower UTI.

**Answer :** We thank the reviewer for this kind comment.

**Major comment 1 :**

However, you conclude that based on the result that postmenopausal age is a risk factor for treatment failure, this risk factor should be considered when considering delayed antibiotic therapy. I do not understand how you came to this part of the conclusion. In delayed antibiotic therapy you do not treat and you rely on spontaneous recovery within a week. In my opinion the risk in delayed therapy is that spontaneous recovery does not happen or that an upper UTI infection develops. Can you elaborate on your conclusion concerning delayed therapy?

**Answer :**

We thank the reviewer for this comment. The definition of delayed antibiotic therapy is correct,^1^ as well as the subsequent risks described, although it should be noted that the risk of progressing to pyelonephritis is in reality quite low (2-5%), as shown in more than one randomized trial.^2,3^ We acknowledge that the ideal study population for the question of delayed therapy would be women with no treatment or with only symptomatic treatment^2^ to truly assess risk factors for non-recovery. However, treated women experiencing treatment failure (either persistence of symptoms or early relapse) essentially become similar to those whose therapy is intentionally delayed, and thus can shed some light, albeit indirectly, on risk factors for non-recovery in either scenario. Effectively, we would want to accurately identify risk factors for persistence or relapse in women who are already on antibiotic therapy; these risk factors should clearly make clinicians wary of attempting delayed therapy in any woman who has them. We have clarified the link between treatment failure and non-recovery in the manuscript.

**Edits in the manuscript:**

p.1, l.41-44: “Postmenopausal age emerged as an independent risk factor for both clinical and microbiological treatment failure in women with lower UTI and should be considered to define women at-risk for non-spontaneous remission, and thus for when considering delayed antibiotic therapy;”

p.2, l.57-59: “As resistance to first-line agents increases and delayed-therapy approaches become more popular, there is a growing need to identify appropriate candidates by identifying risk factors for clinical failure or non-recovery. Fortunately, pyelonephritis remains a rare complication among healthy women.^2,3^”

**Major comment 2 :**

Your study included hospitalized and ambulatory adult women with lower UTI symptoms. I expect delayed therapy to be applied only to ambulatory adult women. Do you know whether your conclusions also apply to ambulatory women only?

**Answer :**

We thank the reviewer for this comment. Currently delayed therapy may concern both outpatient and inpatient women with uncomplicated UTI. However, in the initial clinical trial, 475 (92.6%) of women were outpatient at the time of inclusion, so results can be confidently generalized to the ambulatory setting.

**Major comment 3 :**

An ethical statement is missing.

**Answer :**

We thank the reviewer for this comment. Using only anonymized information (cannot be attributable to initial patients), these data are not subject to the LPD (Loi sur la Protection des Données) and subsequently do not require previous ethical approval by Swiss laws.^4^ However, we have included as statement the IRB approval for the initial clinical trial, p.5, l.125: “The AIDA study was approved by the Geneva Cantonal Ethics Commission (13-014) and Swissmedic (2013DR4095)”.

**Edits in the manuscript:**

- p.5, l.126-127: “Using only anonymized information, this nested study did not require further ethical approval.”

**Major comment 4 :**

It took me a lot of time to understand figure 1, comparing it with the numbers you mention in the Abstract and in Methods. I think it would help if you put all the main groups in the left column and the groups with clinical and microbiological failure on the right, together with the numbers of controls. In Figure 1 it seems like the 340 women with microbiologically confirmed UTI is divided into a group with clinical failure (126 and 214 controls?), a group with microbiological failure (102 with 220 controls) and women with E-coli related UTI (70 with 134 controls?) and 136 women with non-E.coli related UTI.

**Answer :**

We thank the reviewer for this comment. We have revised Figure 1 to improve comparisons with the numbers stated in the manuscript.

**Edits in the manuscript:**

- Update of Figure 1. Study flowchart

Footnote

*: negative or missing cultures at baseline were excluded

**Major comment 5 :**

For the women with E.coli related UTI you only show the women with clinical failure (70 and 134 controls?) but you do not show the women with microbiological failure (71 and 128 controls?). In the Abstract,section Methods you mention that controls were those who did not expoerience clin of microbiol failure.

**Answer :**

We thank the reviewer for this comment. To increase clarity, we have now included women with E. coli-related UTI in the updated flowchart (Figure 1, see comment above). These categories were already detailed in the initial manuscript (p.5, l. 129-135). Controls were defined regarding the case definition (either absence of clinical failure when cases were women with clinical failure, or absence of microbiological failure when cases were women with microbiological failure). This has been further detailed in the methods section.

**Edits in the manuscript:**

- p.4, l.94-98: “Case patients in the nested study were subclassified into “clinical” and “microbiologic”, defined respectively as those who experienced clinical and/or microbiologic failure (as defined previously)^5^ in the 28 days following therapy completion; all available controls were included, defined according to case definitions as women either with clinical or microbiologic success.”

**Major comment 6 :**

In lines 105 and 106 you mention that only women adhering to the RCT's protocol were included. I noticed that cases plus controls not always add up to the total number of women in a main group. In the Result section, I would expect a mention of how many cases or controls were missing because of this.

**Answer :**

We thank the reviewer for this comment. According to the original Figure 1, from the 513 women initially included in the clinical trial, 54 women were excluded either because they had indeterminant results or they did not adhere to the RCT’s protocol. With the improved version of the Figure 1 (see comment above), we can better distinguish cases and controls per population. We effectively recognize that number of cases with microbiological failure and their controls do not match the number of women in main groups. This is because women with missing bacteriological outcomes were excluded. This has now been better detailed in the manuscript, and as a footnote in the Figure 1.

**Edits in the manuscript:**

- p. 4, l.97-98: “all available controls were included, defined according to case definitions as women either with clinical or microbiologic success. Women with missing bacteriological outcomes were excluded.”

**Major comment 7 :**

Line 195: 4 should be in line with the other numbers below 10.

**Answer :**

We thank the reviewer for this comment. We homogenized the wording.

**Edits in the manuscript:**

- p. 9, l.176-178: “Seventeen (9%) women treated with nitrofurantoin and 4 four (2%) treated with fosfomycin had resistant uropathogens; seven (41%) and four (50%) of these had clinical failure and six (35%) and one (25%) microbiologic failure.”

# Reviewer #2

**General comment:**

This study assessed risk factors for clinical and microbiological failure in women with lower UTI via a case-control study nested within a randomized clinical trial. Postmenopausal age was an independent risk factor for clinical and microbiological treatment failure in women with lower UTI; diabetes was not associated with failure. The authors’ assessment of microbiological failure provides an understudied insight that would be of interest to a general urology and primary care audience.

**Answer:**

We thank the reviewer for this comment.

**Major comment 1:**

Abstract and Introduction:

-Given that the authors are from Switzerland, it would be useful for them to identify (either in the abstract of Introduction) whether their cohort – and thus their recommendations – should apply to only a Switzerland demographic, or whether their research could extend outside (or not).

**Answer:**

We thank the reviewer for this comment. Authors are from Switzerland, Poland, and Israel, as are the women included in this study. Therefore, the study results can be generalized to a larger setting. We have specified this in the discussion section.

**Edits in the manuscript:**

- p.11, l.227-229: “Its strengths are its international and relatively large population with a sizeable event rate, improving generalization of findings to other settings, as well as its regular microbiologic follow-up data even among asymptomatic participants.”

**Major comment 2:**

-Similarly, there is some evidence to suggest that women’s menopausal status may also impact of doctor’s choice of therapy and effects of that therapy. The authors do comment on this (and include age as a variable in their analyses), but a more thorough description of this would be useful in describing the demographic, as well as treatment failure (particularly as it relates to age).

**Answer:**

We thank the reviewer for this comment. The effect of menopausal status on therapy’s effectiveness (and thus on possible recommendation of therapy) was a major question of this study. We already described the age characteristics of women as well as treatment failure events relating to age among study participants. Treatment failure occurring among elderly was already discussed in the initial manuscript (p. 10, l.199-205). However, according to the reviewer’s comment, we have also offered a larger perspective of potential benefits by describing the incidence of symptomatic UTI among elderly women.

**Edits in the manuscript:**

- p.10-11, l.206-210: “The influence of age on persistence or relapse might be explained by several factors, including immunosenescence, with its gradual T-cell dysregulation and general deterioration of mucosal immunity, ^6^ and probably by other pathways such as increased urinary stasis and post-menopausal change in the vaginal flora.^7^ Considering both the risk of failure and the high incidence of symptomatic infections among elderly (12.8/100 patient years between 86-90 years),^8^ this population should be considered carefully before delayed antibiotic therapy.”

**Major comment 3:**

Methods:

-The authors state that “only women adhering to the RCT’s protocol were included to avoid the problems of missing data/outcomes and non-adherence” – how many patients were excluded due to this? And was missing data quantified in any way? That is, did most of these patients with missing data have 1-2 missing data points vs. most of their data points missing? And did the demographics of excluded patients differ from those who were included?

**Answer:**

We thank the reviewer for this comment. According to the initial Figure 1, from the 513 women initially included in the clinical trial, 54 women were excluded either because they had indeterminant results or they did not adhere to the RCT’s protocol. With the improved version of the Figure 1 (see comment above), we can better see cases and controls per population. We further described excluded participants in the table below (Table S6 below). Many patients were excluded with undetermined results (43 for clinical failure, and 38 for microbiological failure). Furthermore, excluded patients were more likely to be recruited from Geneva or Tel-Aviv. Otherwise, most of the demographic and clinical characteristics were similar.

Considering missing exposure and outcome (on the 459 included women), we can observe no missing data (Figure below). Importantly, a significant proportion of women did not have cultures in follow-up visits, and thus had missing microbiological outcomes (case 2 is defined as microbiological failure at 28 days). We have included a description of missing data in the results.

Table S6. Population characteristics among included and excluded participants (with undetermined results or not adhering to the initial trial’s protocol)

|  | **Included (n= 459)** | **Excluded (n= 54)** | **P value*** |
| --- | --- | --- | --- |
| Clinical failure (%) | 152 ( 33.1 % ) | 8 ( 72.7 % )** | 0.01 |
| Microbiologic failure (%) | 102 ( 31.7 % ) | 4 ( 25% )** | 0.77 |
| Age (+/-52 years) | 189 ( 41.2 % ) | 18 ( 34 % ) | 0.39 |
| Dysuria (%) | 350 ( 76.3 % ) | 44 ( 83 % ) | 0.35 |
| Frequency (%) | 407 ( 88.7 % ) | 39 ( 73.6 % ) | **<0.01** |
| Urgency (%) | 361 ( 78.6 % ) | 38 ( 71.7 % ) | 0.33 |
| Suprapubic disconfort (%) | 227 ( 49.5 % ) | 38 ( 71.7 % ) | **<0.01** |
| Gross hematuria (%) | 70 ( 15.3 % ) | 12 ( 22.6 % ) | 0.23 |
| Flank pain (%) | 51 ( 11.1 % ) | 10 ( 18.9 % ) | 0.15 |
| Chills (%) | 51 ( 11.1 % ) | 6 ( 11.3 % ) | 1.00 |
| Recurrent UTI (%) | 70 ( 15.3 % ) | 14 ( 25.9 % ) | 0.07 |
| Diabetes Mellitus (%) | 42 ( 9.2 % ) | 3 ( 5.6 % ) | 0.53 |
| Treatment received (%) | 229 ( 49.9 % ) | 29 ( 53.7 % ) | 0.70 |
| Inclusion in Geneva (%) | 159 ( 34.6 % ) | 27 ( 50 % ) | **<0.01** |
| Inclusion in Lodz (%) | 191 ( 41.6 % ) | 9 ( 16.7 % ) | **<0.01** |
| Inclusion in Tel-Aviv (%) | 109 ( 23.7 % ) | 18 ( 33.3 % ) | **<0.01** |
| Risk score for resistance = 0 (%) | 55 ( 12 % ) | 7 ( 13.2 % ) | 0.93 |
| Risk score for resistance = 1 (%) | 326 ( 71 % ) | 36 ( 67.9 % ) |  |
| Risk score for resistance = 2 (%) | 66 ( 14.4 % ) | 9 ( 17 % ) |  |
| Risk score for resistance = 3 (%) | 12 ( 2.6% ) | 1 ( 1.9% ) |  |
| Nitrites on dipstick (%) | 15 ( 3.3 % ) | 2 ( 3.8 % ) | **0.02** |
| Leukocytes on dipstick (%) | 249 ( 54.2 % ) | 34 ( 64.2 % ) |  |
| Both on dipstick (%) | 193 ( 42 % ) | 16 ( 30.2 % ) |  |

*P value by X^2^ test.

** Patients with missing events or undetermined results were excluded

Figure : proportion of missing exposure and outcome among the 459 participating women

**Edits in the manuscript:**

- p.9, l.188: “We had no missing data”
- p.9, l.188: “The 54 participants excluded from this analysis almost shared the same characteristics (Table S6).”
- Table S6 included in the Supplementary Appendix

**Major comment 4:**

-It’s unclear based on the author’s current description why a cutoff of 52 years was chosen and this could use further explanation, particularly given that it is critical to the main results and interpretation.

**Answer:**

We thank the reviewer for this comment. We acknowledge that determination of the cut-off was unclear, line 113 (“The results of this exploratory analysis (supplement) led to a cut-off of 52 years, compatible with the average age of menopause between 50 and 52 years.^9^”). We arbitrary placed the cut-off to 52 years because of physiological consequences from menopausal status,^10^ including immunosenescence, with its gradual T-cell dysregulation and general deterioration of mucosal immunity.^6^ Apart from immunosenescence, age certainly influences both persistence and recurrence of bacteriuria by other pathways such as increased urinary stasis and post-menopausal change in the vaginal flora.^7^

On this basis, the precise cut-off of 52 years was determined graphically (Supplement Figure 1, below) to balance the number of participants in both categories (inferior and superior to 52 years).

This was clarified in the manuscript.

**Edits in the manuscript:**

- p.5, l.111-113: “We defined an age cut-off of 52 years, as the average age of menopause is considered physiologically to be between 50 and 52 years.^9^ The effects of this cut-off can be observed graphically in the supplement (Figure S1).”

**Major comment 5:**

-What statistical analysis software was used?

**Answer :**

We thank the reviewer for this comment. We indicated on p.5, l.122 that all analyses were performed using R.3.6 and RStudio Team (2015), including the associated packages.

**Major comment 6:**

Results:

-Although age was entered based on the 52.5 cutoff, it might be worth classifying women as pre-menopausal or post-menopausal and including that as a variable in the models, if that data was collected by the authors.

**Answer:**

We thank the reviewer for this comment. We agree that true menopause status would be preferred to crude age category. However, we did not collect this data.

**Major comment 7:**

-Table 2 is quite helpful, but it would be useful to see the full models (all variables entered).

**Answer:**

We thank the reviewer for this comment. However, a full model with all variables collected would be at risk of overfitting due to the relatively low number of events. Consequently, we never entered all variables in the models, preferring to enter only pertinent pre-selected variables in parsimonious simple models. The selection of variables is described in lines 118-121, based on literature and Bayesian Model Averaging methods.

**Major comment 8:**

Discussion:

-The authors note that recruitment site (and variation among centres) impact clinical outcomes. Why was this the case? Additional explanation is needed for results to be interpretable.

**Answer:**

We thank the reviewer for this comment. A centre effect may have influenced clinical outcomes, with participants from Tel-Aviv potentially more likely to suffer from clinical failure. Of note, this centre effect waned when performing the same analysis for both clinical and microbiological failure among participants with only E.coli isolated (and not mixed flora).

When comparing exposures between women from Tel-Aviv compared to other centres (table below), we observed more symptomatic women with more severe symptoms (flank pain, chills, recurrent UTIs, …). We could thus hypothesise that women included in this centre had more severe illnesses, impacting clinical outcomes.

Another hypothesis, however, is that the difference would be explained by cultural differences, with heterogeneous pain thresholds impacting detection of clinical failure and potentially generating a reporting bias. Such an observation has already been reported in a previous study among participants receiving the recombinant vesicular stomatitis virus-vectored Zaire Ebola (rVSV-ZEBOV) vaccine in Geneva (Switzerland), and in Lambaréné (Gabon). Participants from Lambaréné reported fewer and milder adverse events (subjective fever, myalgia, chills, etc.) compared to Geneva participants. Yet the vaccine’s signature (plasma chemokines/cytokines) was essentially the same in both populations, reflecting a lower threshold to report more (and more severe) events in the Geneva population.^11^

Table. Population characteristics among participants from Tel Aviv versus other centers

|  | Participants from Tel-Aviv (n= 109) | Participants from Geneva and Lodz (n= 350) | p-value |
| --- | --- | --- | --- |
| Age (+/-52 years) | 37 ( 33.9 % ) | 152 ( 43.4 % ) | 0.10 |
| Dysuria (%) | 84 ( 77.1 % ) | 266 ( 76 % ) | 0.92 |
| **Frequency (%)** | 89 ( 81.7 % ) | 318 ( 90.9 % ) | **0.01** |
| Urgency (%) | 89 ( 81.7 % ) | 272 ( 77.7 % ) | 0.46 |
| **Suprapubic disconfort (%)** | 87 ( 79.8 % ) | 140 ( 40 % ) | **<0.001** |
| **Gross hematuria (%)** | 29 ( 26.6 % ) | 41 ( 11.7 % ) | **<0.001** |
| **Flank pain (%)** | 24 ( 22 % ) | 27 ( 7.7 % ) | **<0.001** |
| **Chills (%)** | 30 ( 27.5 % ) | 21 ( 6 % ) | **<0.001** |
| **Reccurent UTI (%)** | 34 ( 31.2 % ) | 36 ( 10.3 % ) | **<0.001** |
| Diabetes Mellitus (%) | 10 ( 9.2 % ) | 32 ( 9.1 % ) | 1 |
| Treatment received (%) | 57 ( 52.3 % ) | 172 ( 49.1 % ) | 0.64 |
| **Risk score for resistance = 0 (%)** | 0 ( 0 % ) | 55 ( 15.7 % ) | **<0.001** |
| **Risk score for resistance = 1 (%)** | 104 ( 95.4 % ) | 222 ( 63.4 % ) | **<0.001** |
| **Risk score for resistance = 2 (%)** | 5 ( 4.6 % ) | 61 ( 17.4 % ) | **<0.001** |
| **Risk score for resistance = 3 (%)** | 0 ( 0 % ) | 12 ( 3.4 % ) | **<0.001** |
| **Nitrites on dipstick (%)** | 2 ( 1.8 % ) | 13 ( 3.7 % ) | **<0.001** |
| **Leukocytes on dipstick (%)** | 90 ( 82.6 % ) | 159 ( 45.4 % ) | **<0.001** |
| **Both on dipstick (%)** | 17 ( 15.6 % ) | 176 ( 50.3 % ) | **<0.001** |

**Edits in the manuscript:**

- P.11, l.221-224: “An increased proportion of clinical (but not bacteriologic) failure was observed among Tel Aviv participants. This centre effect could be explained by the inclusion of women with more severe illness, or by cultural differences in the self-reporting of symptoms, possibly generating a reporting bias, as observed elsewhere.[34]”

**Major comment 9 :**

-Given that the finding that diabetes was strongly associated with microbiological failure, more discussion should be included. Was this predicted/hypothesized? If yes, why or why not?

**Answer:**

We thank the reviewer for this comment. The association between diabetes and treatment failure was effectively hypothesized. As described in the initial manuscript, diabetes is generally associated with more frequent UTI, primarily through immunological impairment, neurogenic bladder, and increased bladder glucose concentration.^12^ Furthermore, other studies observed an association between glycemic control and asymptomatic bacteriuria.^13,14^ This had been detailed in the manuscript.

**Edits in the manuscript:**

- p.11, l.218-220: “The association between diabetes and microbiological failure was thus expected, with association between glycemic control and asymptomatic bacteriuria already observed elsewhere.^13,14^”

**Major comment 10:**

-When discussing limitations, the authors note that strength of their study was that it was multi-centre; however, these centres clearly varied (given recruitment site impacted outcomes). Why might that be more of a limitation? – the authors might want to discuss this a bit more.

**Answer :**

We thank the reviewer for this comment. Effectively, there may be a centre effect impacting outcome, which is often expected in multicenter studies. This variation is well addressed by statistical adjustment, and benefits the study by improving the generalization of findings.

**Edits in the manuscript:**

- P.11, l.221-224: “An increased proportion of clinical (but not bacteriologic) failure was observed among Tel Aviv participants. This potential centre effect could be explained by the inclusion of women with more severe illness, or by cultural differences in the self-reporting of symptoms, possibly generating reporting bias, as observed elsewhere.[34]”
- p.112, l.227-229: “Its strengths are its international and relatively large population with a sizeable event rate, improving generalization of findings to other settings”

**REFERENCES**

1.	de la Poza Abad M, Mas Dalmau G, Gich Saladich I, Martínez García L, Llor C, Alonso-Coello P. Use of delayed antibiotic prescription in primary care: a cross-sectional study. *BMC Fam Pract*. 2019;20(1):45. doi:10.1186/s12875-019-0934-7

2.	Kronenberg A, Bütikofer L, Odutayo A, et al. Symptomatic treatment of uncomplicated lower urinary tract infections in the ambulatory setting: randomised, double blind trial. *BMJ*. 2017;359:j4784. doi:10.1136/bmj.j4784

3.	Gágyor I, Bleidorn J, Kochen MM, Schmiemann G, Wegscheider K, Hummers-Pradier E. Ibuprofen versus fosfomycin for uncomplicated urinary tract infection in women: randomised controlled trial. *BMJ*. 2015;351:h6544. doi:10.1136/bmj.h6544

4.	Préposé fédéral à la protection des données et à la transparence. Protection des données et recherche en général. Accessed June 14, 2021. https://www.edoeb.admin.ch/edoeb/fr/home/datenschutz/statistik--register-und-forschung/forschung/datenschutz-und-forschung-im-allgemeinen.html

5.	Huttner A, Kowalczyk A, Turjeman A, et al. Effect of 5-Day Nitrofurantoin vs Single-Dose Fosfomycin on Clinical Resolution of Uncomplicated Lower Urinary Tract Infection in Women: A Randomized Clinical Trial. *JAMA*. 2018;319(17):1781-1789. doi:10.1001/jama.2018.3627

6.	Gavazzi G, Krause K-H. Ageing and infection. *Lancet Infect Dis*. 2002;2(11):659-666. doi:10.1016/s1473-3099(02)00437-1

7.	Liang SY, Mackowiak PA. Infections in the Elderly. *Clin Geriatr Med*. 2007;23(2):441-456. doi:10.1016/j.cger.2007.01.010

8.	Nicolle LE. Urinary Tract Infections in the Older Adult. *Clin Geriatr Med*. 2016;32(3):523-538. doi:10.1016/j.cger.2016.03.002

9.	Nelson HD. Menopause. *The Lancet*. 2008;371(9614):760-770. doi:10.1016/S0140-6736(08)60346-3

10.	Foxman null. Urinary Tract Infection in Postmenopausal Women. *Curr Infect Dis Rep*. 1999;1(4):367-370. doi:10.1007/s11908-999-0043-1

11.	Huttner A, Combescure C, Grillet S, et al. A dose-dependent plasma signature of the safety and immunogenicity of the rVSV-Ebola vaccine in Europe and Africa. *Sci Transl Med*. 2017;9(385):eaaj1701. doi:10.1126/scitranslmed.aaj1701

12.	Boyko EJ, Fihn SD, Scholes D, Chen C-L, Normand EH, Yarbro P. Diabetes and the risk of acute urinary tract infection among postmenopausal women. *Diabetes Care*. 2002;25(10):1778-1783. doi:10.2337/diacare.25.10.1778

13.	Bharti A, Chawla SPS, Kumar S, et al. Asymptomatic bacteriuria among the patients of type 2 diabetes mellitus. *J Fam Med Prim Care*. 2019;8(2):539-543. doi:10.4103/jfmpc.jfmpc_403_18

14.	Renko M, Tapanainen P, Tossavainen P, Pokka T, Uhari M. Meta-analysis of the significance of asymptomatic bacteriuria in diabetes. *Diabetes Care*. 2011;34(1):230-235. doi:10.2337/dc10-0421
